# Stimulation maps: visualization of results of quantitative intraoperative testing for deep brain stimulation surgery

**DOI:** 10.1007/s11517-020-02130-y

**Published:** 2020-01-30

**Authors:** Ashesh Shah, Dorian Vogel, Fabiola Alonso, Jean-Jacques Lemaire, Daniela Pison, Jérôme Coste, Karin Wårdell, Erik Schkommodau, Simone Hemm

**Affiliations:** 1 Institute for Medical Engineering and Medical Informatics, University of Applied Sciences and Arts Northwestern Switzerland, Muttenz, Switzerland; 2 Department of Biomedical Engineering, Linköping University, Linköping, Sweden; 3 CNRS, SIGMA Clermont, Institut Pascal, Université Clermont Auvergne, Clermont-Ferrand, France; 4 Service de Neurochirurgie, Hôpital Gabriel-Montpied, Centre Hospitalier Universitaire de Clermont-Ferrand, Clermont-Ferrand, France

**Keywords:** Deep brain stimulation, Electric field simulations, Accelerometry, Data visualization, Essential tremor

## Abstract

Deep brain stimulation (DBS) is an established therapy for movement disorders such as essential tremor (ET). Positioning of the DBS lead in the patient’s brain is crucial for effective treatment. Extensive evaluations of improvement and adverse effects of stimulation at different positions for various current amplitudes are performed intraoperatively. However, to choose the optimal position of the lead, the information has to be “mentally” visualized and analyzed. This paper introduces a new technique called “stimulation maps,” which summarizes and visualizes the high amount of relevant data with the aim to assist in identifying the optimal DBS lead position. It combines three methods: outlines of the relevant anatomical structures, quantitative symptom evaluation, and patient-specific electric field simulations. Through this combination, each voxel in the stimulation region is assigned one value of symptom improvement, resulting in the division of stimulation region into areas with different improvement levels. This technique was applied retrospectively to five ET patients in the University Hospital in Clermont-Ferrand, France. Apart from identifying the optimal implant position, the resultant nine maps show that the highest improvement region is frequently in the posterior subthalamic area. The results demonstrate the utility of the stimulation maps in identifying the optimal implant position.

**Figure:**
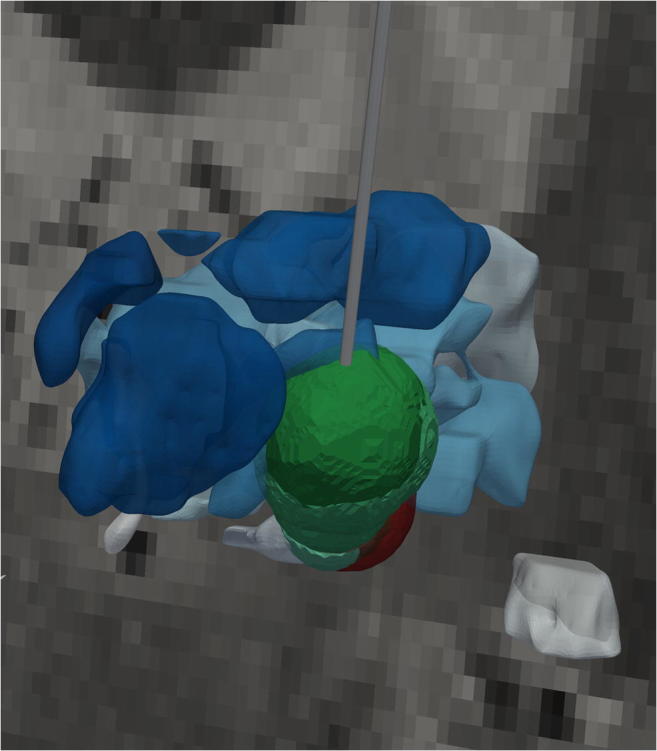
Graphical abstract

Graphical abstract

## Introduction

Deep brain stimulation (DBS) is a neurosurgical treatment for movement disorders like essential tremor (ET). Patients undergo a complex surgical procedure to implant leads in the brain, which are continuously stimulated through a subcutaneously implanted pulse generator (IPG). The outcome significantly depends on the location of the DBS lead in the brain. Over the years of DBS usage, clinicians have established few specific, disease-dependent target regions, e.g., the ventro-intermedius nucleus (VIM) of the thalamus for ET. However, as these targets have a size in the range of millimeters and as the exact mechanisms behind the functioning of DBS are still incompletely known [24], most clinicians still implant the DBS lead after testing various positions on locally anesthetized patients during surgery [1, 22].

Before the actual surgery, clinicians perform planning using specially designed software to identify the target structure on the patient’s anatomical images and the best path to reach it from an entry point in the skull. During the surgery, one or more parallel test electrodes are inserted along the planned path and neuronal recording and stimulation tests are performed at pre-determined positions. Therapeutic and adverse effects are evaluated at these stimulation test positions. The details of the surgical procedure may vary between centers, but certain limitations are observed in all centers: the therapeutic effects of stimulation tests, for example, are evaluated visually or through passive movements using subjective clinical scales [3–5]. Further, after completing stimulation tests for one hemisphere, the surgical team has to “mentally” visualize the results in relation to the anatomy to identify the optimal implant position.

We have previously published a method [42] using accelerometers to quantitatively evaluate improvement in tremor during intraoperative stimulation tests. By classifying the data collected from DBS for ET patients based on the position of the electrode with respect to the nuclei, it was possible to show that the ventro-oral (VO) nucleus of the thalamus can be as efficient in reducing tremor as the VIM [41]. However, the effect of stimulation spreads farther in the region surrounding the electrode depending on the brain tissue. In order to estimate this spatial effect of stimulation, a technique to simulate the patient-specific electric field (EF) distribution [3] was adapted for intraoperative stimulation tests. The collective analysis of the application of this technique to 272 stimulations showed that reduction in tremor correlates with increased interaction of the electric field with VIM and the prelemniscal radiations (PLR) [23].

In comparison to collective data analysis in our previous studies, we propose in the current paper a method to visually analyze the high amount of data that is generated per patient. The aim is to combine the patient-specific electric field simulations with tremor improvement quantified through accelerometry as well as stimulation-induced adverse effects, and to visually present them in the form of so-called stimulation maps superimposed to the patient-specific anatomy to assist in surgical decision making, i.e., choosing the optimal implant position for the chronic DBS lead for the given patient. This stimulation map approach has been applied retrospectively to 9 DBS lead implantations from Clermont-Ferrand University Hospital in France to illustrate its advantage over current methods.

## Method

### Surgical procedure

The routine surgical procedure at the University Hospital in Clermont-Ferrand began with a meticulous pre-surgical planning. A brief description of the procedure is given here while a complete description can be found elsewhere [46]. A stereotactic CT (0.59 × 0.59 × 1.25 mm), stereotactic T1 MRI (0.63 × 0.63 × 1.30 mm), and white-matter attenuation inversion recovery (WAIR, 0.54 × 0.53 × 2.0 mm) sequence were acquired (Sonata 1.5T, Siemens, Germany) to be used for the planning. Based on the spontaneous contrast observed on the WAIR sequence and an in-house developed high field (4.7 Tesla) brain atlas, the neurosurgeon carefully outlined various thalamic nuclei and basal ganglia structures using a commercial planning software iPlan stereotaxy 3.0 (Brainlab). After delineating the desired target structure, i.e., the VIM for ET patients, two parallel trajectories were planned from an entry point in the skull. The trajectories usually follow the path from the superior-anterior-lateral thalamus (VO) towards the inferior-posterior-medial direction passing the VIM with a target at its inferior border. Various test stimulation positions (between 5 and 10) were planned along each trajectory spanning the whole region of interest.

During surgery, the stereotactic co-ordinates of the planned trajectories obtained from the planning software were set up on the Leksell Stereotactic System (Elekta, Stockholm, Sweden) using the repositioning kit. Two intraoperative exploratory electrodes were inserted along the previously identified trajectories. Micro-electrode recording (MER) was performed at all the planned test-stimulation positions along both trajectories simultaneously to confirm the location of the trajectories in relation to the surrounding anatomical structures [12]. Stimulation tests were then administered at these positions sequentially, with stimulation current varied in most cases from 0 to 3 mA in steps of 0.2 mA. Other stimulation parameters, i.e., mono-polar stimulation of 60 μs duration at a frequency of 130 Hz [30] remained the same for all positions. The highest visually observed improvement in tremor was noted along with the corresponding stimulation current amplitude for every test position. The amplitudes resulting in adverse effects, if any, were also noted.

After completion of stimulation tests for one brain hemisphere, the surgical team compared the results (current amplitudes improving tremor and/or inducing adverse effect) and mentally visualized the information in relation to the patient’s anatomy. The DBS lead (Medtronic 3389, Medtronic, Minneapolis, USA) was implanted at a position along one of the tested trajectories fulfilling the following conditions:

- Low therapeutic stimulation current amplitude
- High threshold for stimulation-induced adverse effects
- Neighboring test positions having relatively low therapeutic stimulation current amplitudes
- Anatomical position

### Accelerometric tremor evaluation

The changes in tremor during surgery were evaluated applying a previously published method using an accelerometer [42]. In short, a 3-axis acceleration sensor was attached to the patient’s wrist and data were recorded and stored for offline analysis using an in-house developed computer application. Data recording was synchronized with the MicroGuide Pro electrophysiology system (Alpha Omega Eng., Nazareth, Israel) used for MER and stimulation tests. For every stimulation test, changes in tremor were determined compared with the data recorded immediately before the start of the test (baseline state). By using such a protocol, changes in tremor due to brain tissue damage caused by introducing the electrode would not influence the quantitative assessment.

Post-operative analysis of the data using Matlab consisted of calculating and filtering the magnitude of acceleration. Outcome measures (standard deviation, signal energy, and amplitude of dominant frequency) which have been shown to correlate with clinical changes [42] were extracted in a windowed manner (time length of 2 s). The outcome measures for the stimulation data were normalized to the corresponding outcome measures of the baseline. The quantitative improvement in tremor was expressed by the mean of the three normalized outcome measures for each window, and the average improvement per stimulation current amplitude was determined.

### Spatial distribution of stimulation

The effects of electrical stimulation in the brain are not confined to the location of the electrode, but spread farther into the brain in all directions depending on the stimulation parameters and the brain tissue surrounding the contact. To understand these spatial effects of the stimulation, an established patient-specific FEM-modeling technique for DBS leads [3, 4, 49] was adapted to the conditions and to the setup of intraoperative stimulation tests, and the distribution of the EF around the electrodes within the brain was simulated. The simulation method is briefly described below and details can be found in a previous publication [23].

#### Microelectrode model

A model of the microelectrode (Neuroprobe, Alpha Omega Engineering) used in Clermont-Ferrand University Hospital was developed with its specific dimensions. As electrophysiological evaluations were performed through two parallel electrodes, a second model of the MER-electrode was positioned at a distance of 2 mm. The ends of the grounded guide tubes were fixed at 12 mm above the target point. In consequence, the distance (d) between the guide tube and the middle of the stimulating contact decreased or increased respectively when the simulation site was before or after the target (12 mm ± d) along the trajectory.

#### Patient-specific brain model

Patient-specific brain models [3, 5] were created to perform patient-specific simulations of the electric field distribution. The model is a cuboid region of interest of approximately 100 mm encompassing the thalamus consisting of a matrix of electrical conductivities obtained from the patient images. An in-house developed software (ELMA 2.3; [47]) was used for creating brain tissue models. T1 images were segmented according to the intensity values into CSF, gray and white matter, and blood [2]. The segmented image voxels were assigned with the electrical conductivity values (σ)^1^ based on published literature [19, 48] taking into account the specific frequency and pulse width values: CSF-2.0 Siemens/m (S/m), blood-0.7 S/m, gray matter-0.123 S/m, and white matter 0.075 S/m. Conductivity values for voxels between the thresholds were linearly interpolated.

#### Patient-specific stimulation data

**Fig. 1:**
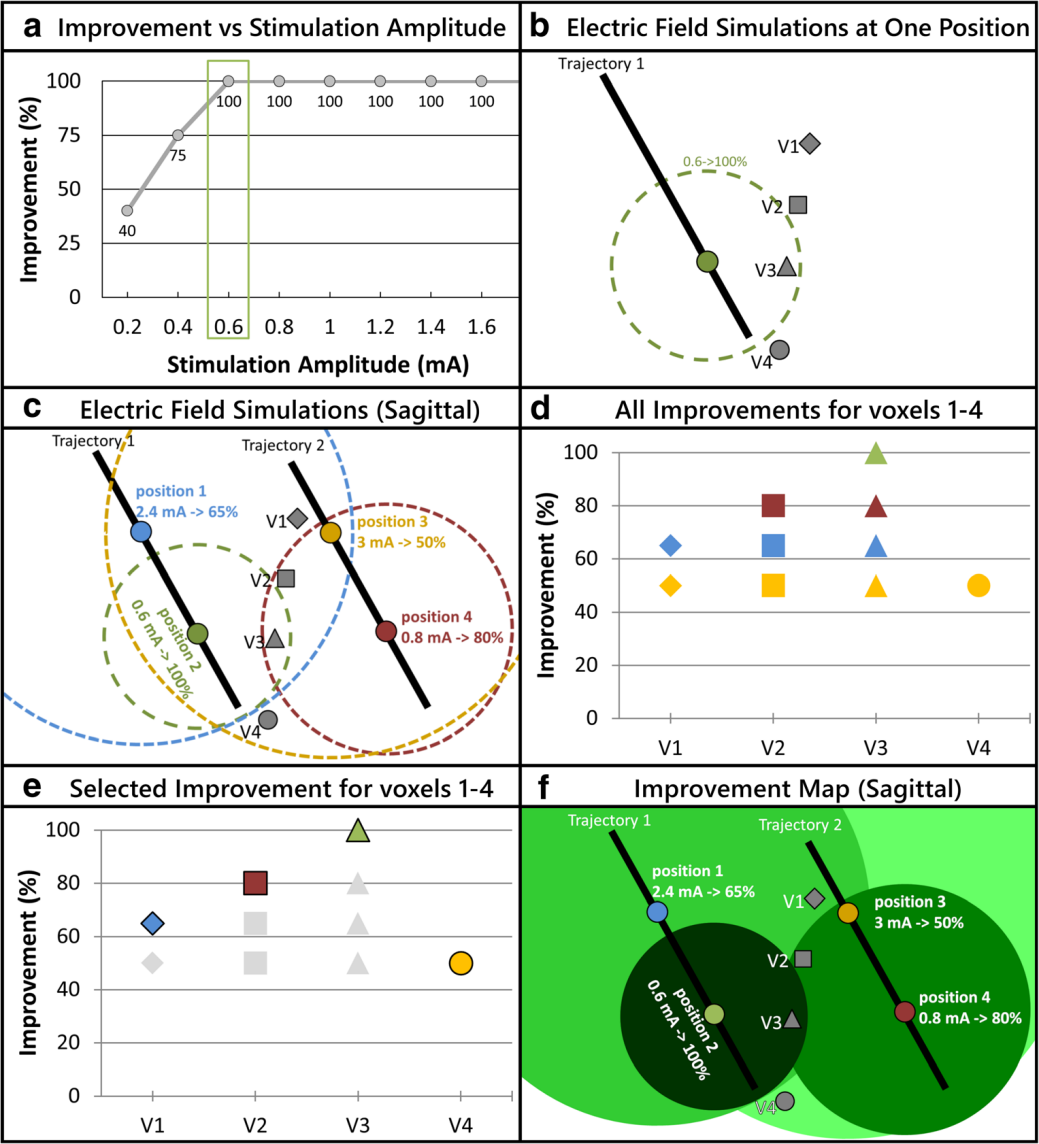
Diagrammatic representation of the improvement maps approach. **a** Graph showing improvement vs stimulation current amplitude for one test position. The green rectangle highlights the lowest amplitude resulting in the highest tremor improvement for this test position. **b** EF simulation for the chosen current amplitude and the respective tremor improvement at the test position shown in part a. **c** EF simulations of the lowest amplitude resulting in highest tremor improvement as in part b, at 4 different positions on two parallel trajectories. **d** Graph showing the improvement values that can be associated to voxels V1 to V4 based on the different EFs they are enclosed in, as depicted in part c. **e** Graphical representation of the chosen improvement value assigned to voxels V1 to V4. **f** Improvement map that results after assigning the selected improvement from part e (e.g., maximum) to all the voxels in the stimulation test region

**Fig. 2:**
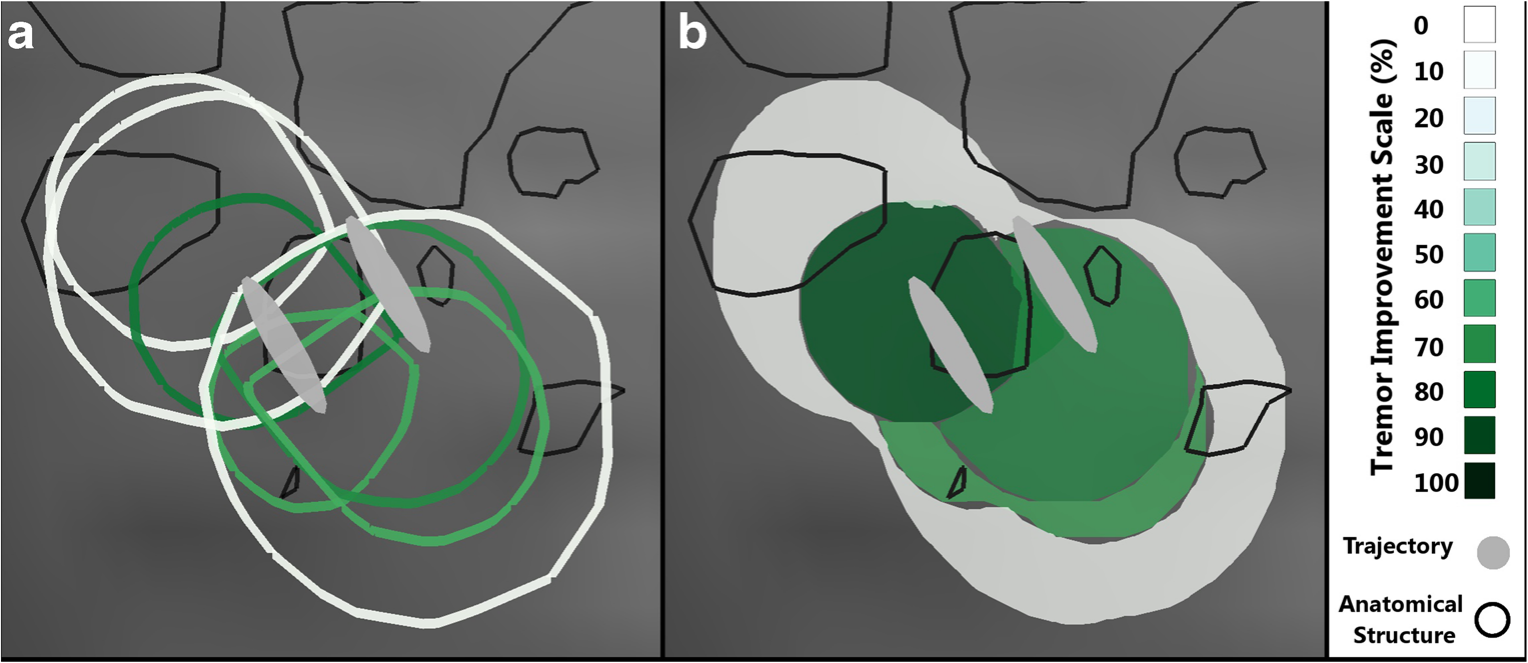
Sagittal view of electric field simulations for left hemisphere of patient 5. The black outline represents different thalamic nuclei and the gray ovals are the projections of the electrode’s trajectories. **a** Visualization of the outlines of 8 EFs. The color of the border represents the improvement in tremor. It underlines the need to summarize the information using improvement maps. **b** Maximum improvement map of the EFs seen in **a**. The shade of green corresponds to the improvement associated with the region based on the scale in the legend

For each implantation, the planned trajectory to reach the target structure and the stimulation test positions along the trajectory were extracted from the planning software (iPlan stereotaxy) and converted to the co-ordinate system of the brain model using Matlab. The target co-ordinates and the trajectory angles were used to calculate and to place the stimulating contact and the parallel electrode in the brain model for the different stimulation test positions. The stimulation current amplitude applied intraoperatively was used to simulate the EF. In order to keep the simulation time reasonable, we decided to simulate for each stimulation test position the EF distribution for the current amplitudes with (1) the first appearance of *highest* change in tremor (between 0.2 and 3 mA, Fig. 1a, b) and (2) the first appearance of adverse effects (between 1 and 5 mA).

#### Electric field simulations

The spatial distribution of EF was simulated by using the equation of continuity for steady state current:

1 $$ \nabla .\overset{\acute{\mkern6mu}}{J}=\nabla .\left(\sigma \nabla V\right)=0 $$

where J is the current density, σ is the scalar volume of the patient-specific electrical conductivity values for the region of interest (thalamus and its neighborhood), and V is the electric potential. After placing the electrodes at a desired stimulation test position, the active contact was set to the current amplitude described in Section 2.3.3, and the guide tube was set to ground, resulting in a monopolar configuration. The inactive contact of the parallel lead was set to floating potential and the exterior boundaries of the tissue model were set to electrical insulation. The in-built mesh generator (Comsol Multiphysics 5.2, Comsol AB, Sweden) defined the mesh density (approximately 250,000 tetrahedral elements) where the smallest elements (0.03 mm) were located close to the stimulating contacts in order to capture the strong EF gradients. Previous research has shown that the EF isolevel of 0.2 V/mm represents the neuronal activation for the thalamic region [6, 28]. Therefore, the Cartesian co-ordinates of the surface of EF volume for 0.2 V/mm were exported for further analysis.

#### Surgical planning data

In addition to the planned trajectory and target information, the manually outlined anatomical structures were extracted from the iPlan software via a specifically designed interface based on VVLink and VTK (VTK 5.2.0, Kitware Inc. New York, USA). In order to reduce error sources, only the CT data set was used as reference for the outlined anatomical structures and the target co-ordinates. In consequence, the structures initially outlined on the WAIR-weighted sequence were resampled in iPlan to the stereotactic CT data set providing a higher resolution and minimal distortion of the stereotactic reference system.

### Clinical application

A clinical study was undertaken at the University Hospital in Clermont-Ferrand, France (Ref: 2011-A00774-37/AU905, Comité de Protection des Personnes Sud-Est 6, Clermont-Ferrand, France), and 5 patients who were treated for ET using DBS were included for the current work (Table 1) after obtaining informed written consent. The number of stimulation tests varied from patient to patient based on the size of the region of interest and the occurrence of adverse effects during the stimulation tests. Three types of adverse effects were observed for different patients, i.e., pyramidal effects, paresthesia, and dysarthria. Accelerometer data were recorded during the surgery following the above presented protocol to evaluate the changes in tremor induced by varying stimulation current amplitudes. For patient 1, data acquired during the implantation of the left hemisphere were excluded from the analysis due to lack of synchronization with the electrophysiological data (recording software error).

**Table 1:** Surgical details of the patients participating in the clinical study

Patient number	Brain side	Trajectory	Number of test positions	Number of electric field simulations	Side effects
1	Right	Central	7	7	
		Posterior	8	8	
2	Left	Central	5	8	Paresthesia, pyramidal effect
		Posterior	5	7	
2	Right	Central	7	8	Paresthesia
		Posterior	7	8	
3	Left	Central	8	8	
		Posterior	8	8	
3	Right	Central	8	8	Paresthesia
		Posterior	8	10	
4	Left	Central	9	9	
		Posterior	9	9	
4	Right	Central	5	9	Paresthesia, pyramidal effect
		Posterior	5	9	
5	Left	Central	8	8	Dysarthria
		Posterior	8	9	
5	Right	Central	8	8	Dysarthria
		Posterior	8	9	

### Stimulation maps

The goal of the intraoperative stimulation tests is to determine the optimal position for implanting the chronic stimulation lead (see Section 2.1). In current clinical practice, this decision is made using paper–pencil notes and mental visualization of a large amount of stimulation test results: for one implantation, on average, there are 2 trajectories with 7 different positions (Table 1). The aim of the visualization of all these data is to support in identifying the region that results in the highest improvement and that is far away from adverse effect-inducing regions. Therefore, the stimulation maps consist of two subparts: (a) improvement maps visualizing the therapeutic effect of stimulation and (b) adverse effect maps visualizing the intraoperative adverse effects of stimulation.

#### Improvement maps

In order to visualize the therapeutic effect of intraoperative stimulation tests, in the present approach, we have chosen for simulation, out of the multitude of available amplitudes in a position (Fig. 1a), the one which is considered for the decision-making process: the lowest amplitude resulting in the highest improvement in symptoms (Fig. 1b). The combined visualization of the different EF simulations for one implantation results in overlapping EFs, each of which corresponds to a specific clinical improvement. In consequence, many voxels can be encompassed in more than one EF simulation (Fig. 1c), i.e., associated to multiple improvement values (Fig. 1d). The proposed solution is to associate only one improvement value to each voxel in the test stimulation region (Fig. 1e) to summarize the data and to facilitate analysis (Fig. 1f).

For a predetermined goal of visualization, various types of improvement maps can be created by choosing an improvement value that is assigned to a given voxel. On closer examination of the EF simulations of the patients (Fig. 2a), it was observed that the (smaller) EF simulations of low current resulting in high tremor improvement (green outlines) at one position were encompassed by (larger) simulations at neighboring positions of higher current with low tremor improvement (white outlines). Based on this observation and as the goal is to identify the region within which the highest improvement could be observed during the tests, each voxel was assigned with the maximum value of improvement out of the existing values (“maximum improvement map”, Fig. 2b). Although one improvement value is assigned to each individual voxel, it should be considered that the exclusive stimulation of a collective group of voxels results in the improvement in tremor depicted in the maps.

#### Adverse effect maps

The adverse effect maps approach has been presented previously [44]. As described in Section 2.3.3, patient-specific EF was simulated for the lowest current amplitudes that resulted in any adverse effects. These amplitudes represent the upper limits of safe stimulation. Therefore, these simulations are visualized as outer borders of therapeutic stimulation. During one implantation, if the same adverse effect was observed at more than one position, the EF simulations were combined to form one region representing the particular adverse effect.

#### Implementation

**Fig.3:**
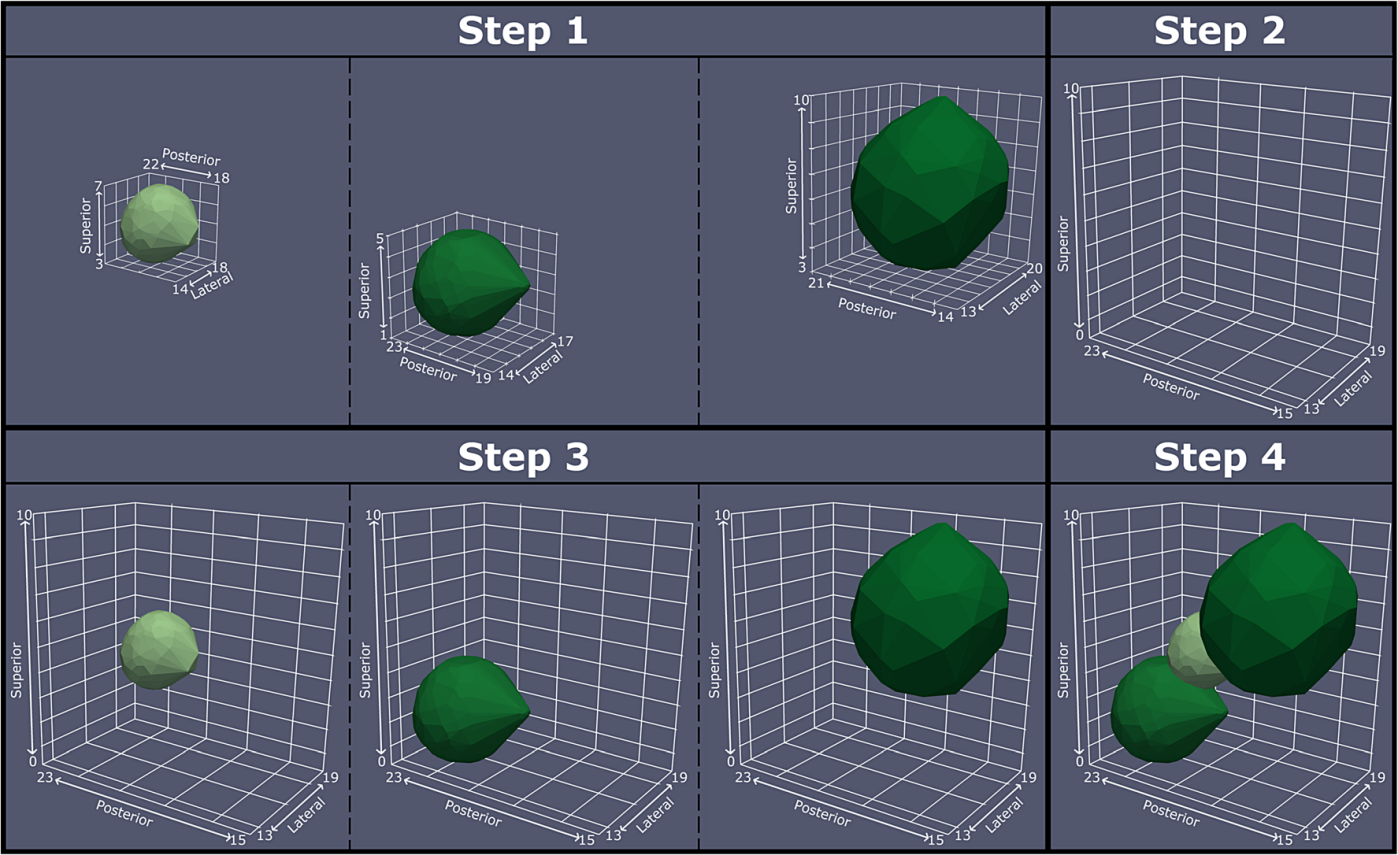
A diagrammatic representation of the steps to generate the improvement maps. The individual EF simulations are imported (Step 1) and the size of the stimulation test region is calculated. A mesh grid of this size with 4 times the resolution of the CT data is created (Step 2). The location of each EF simulation in this mesh grid is determined (Step 3). Each voxel in the improvement map is then assigned with the improvement value based on the chosen mathematical function (Step 4)

From the EF simulations, the improvement map is generated using Matlab scripts as presented in Fig. 3. All the EF simulation files from COMSOL (refer Section 2.3.4) are imported in Matlab, and the extent of the stimulation test region in a given hemisphere is calculated (Fig. 3, Step 1). A 3D mesh-grid with a resolution 4 times the resolution of the CT images is created (Fig. 3, Step 2). Using the Delaunay Triangulation (Boris Delaunay 1934) and identifying points in the triangulation, the position of each EF in this mesh-grid is identified and a mask is created. The mask is multiplied with the improvement associated with the EF, i.e., the voxels within the EF hold the improvement value and the rest are set to zero. All such improvement masks have the same dimensions and are therefore appended together to create a 4D matrix (Fig. 3, Step 3). By using a many to one function (e.g., minimum, mean, maximum, etc.) on the 4th dimension, a 3D matrix is obtained having the same dimensions as the mesh-grid, and each voxel holding only one improvement out of all the EF simulations that encompassed it (Fig. 3, Step 4). After identifying the different levels of improvement in this 3D matrix and using the location-query function, the X, Y, and Z co-ordinates of each improvement level are obtained and exported. For the EF of adverse effect thresholds, their co-ordinates in the mesh-grid are identified and exported as csv files without any additional processing.

For visual analysis of the stimulation map, data were imported in Paraview (Kitware, Clifton Park, NY, USA) using Python (Python.org) scripts in the following order: (1) the T1 MR and the WAIR MR image data sets were imported and cropped to the region of interest. (2) The csv files containing the co-ordinates of the thalamic nuclei (see Section 2.3.5) were imported one by one, and visualized as surface extracts of Delaunay triangulation of these co-ordinates. (3) The csv files containing the improvement map were imported and visualized as Delaunay triangulations. (4) The simulations for adverse effect thresholds were imported and visualized as surface extracts of Delaunay triangulations to depict them as boundary beyond which adverse effects were observed. The data were first visualized in the 3D view and then loaded into the orthographic slice view using a custom macro script.

### Data analysis

The stimulation maps were analyzed individually by simultaneously moving the axial, coronal, and sagittal sections across all the visualized data in the Paraview’s orthographic slice view. For each map, the location of the region showing the highest improvement was identified and carefully studied with respect to the outlined anatomical structures and the adverse effect threshold outline. The trajectory and optimal depth that would have been selected based on the stimulation maps to implant the lowest contact of the permanent lead was determined such that the lower border of this contact would align with the lower edge of the highest improvement region. This would allow stimulation of the highest improvement region through a mono-polar or bi-polar setup of the lead. In addition, in each map, the interaction of various anatomical structures with the induced clinical effects was meticulously examined. In the highest improvement regions, structures partially or completely covered were identified. For adverse effects, structures touching the adverse effect threshold outlines and external to any improvement region were noted. Finally, recurring structures were studied for their interaction with the highest improvement region and the adverse effect threshold outline across all the implantations.

## Results

Through the clinical study in the University Hospital in Clermont-Ferrand, accelerometer data was recorded for a total of 129 test stimulation positions from the 5 DBS surgeries. Using the FEM-based technique, 148 simulations (129 therapeutic + 19 adverse effects) were performed based on these stimulation tests and summarized in 9 stimulation maps. These maps vary from patient to patient depending on the planned trajectory and the test stimulation positions along it.

**Fig. 4:**
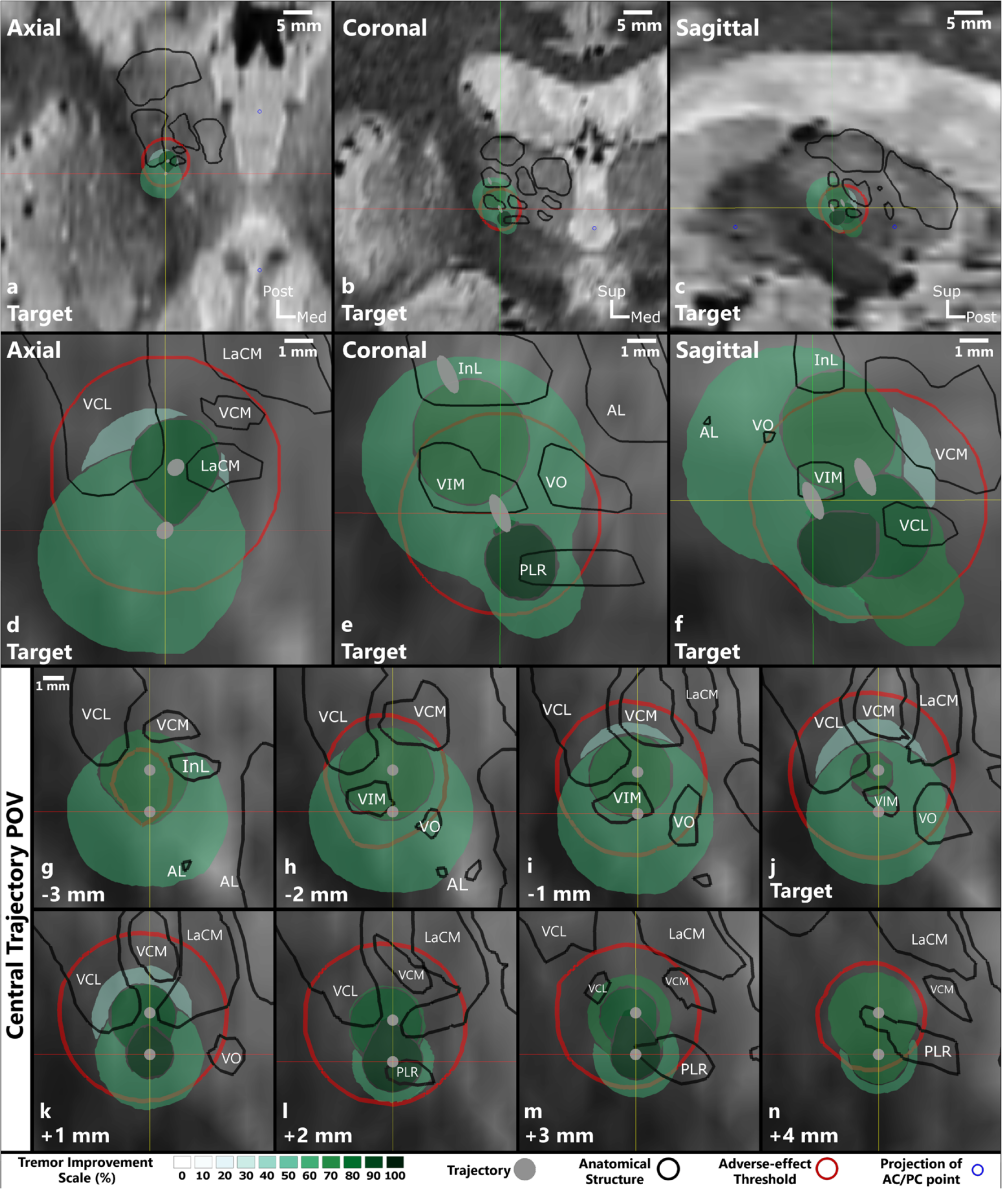
Images of the stimulation map for the right hemisphere of patient 5. Parts **a** to **c** show the overview of the explored region in relation to the patient’s brain in the form of orthogonal slices at the target position. Parts **d** to **f** show a close-up (magnification of 5) of the stimulation maps at the same position. Parts **g** to **n** are views along the central trajectory at the different stimulation test positions from − 3 to + 4 mm. The bottom part of the figure shows the legend containing the improvement scale in shades of green, representation of the trajectory in gray, outline of anatomical structures in black, side-effect outline in red, and projection of AC/PC point in blue. The names of the relevant thalamic nuclei are abbreviated based on the nomenclature in Lemaire et al. (2010) as follows: VIM ventrointermediate, VCL ventrocaudal lateral, VCM ventrocaudal medial, InL intermedio-lateral, LaCM laminar caudal medial, VO ventro oral, PLR prelemniscal radiations

Figure 4 shows a typical stimulation map of the right hemisphere of patient 5. The inferolateral part of the thalamus was explored during the stimulation tests (Fig. 4a–c) with the planned target at the inferior border of the VIM (Fig. 4d–f). The highest improvement region (95%) can be seen further along the trajectory from + 1 mm to + 3 mm in front of the target (Fig. 4e, f and k–m) shaped like a drop with its peak in the posterior direction. The region starts just below the VIM, touches the medial edge of the ventrocaudal lateral nucleus (VCL), lateral edge of the center median nucleus (LaCM), and penetrates the supero-lateral part of the PLR (Table 2, row 17). On the other hand, the spherical adverse effect (dysarthria) outline (red) interacts with VO, VCL, VCM, LaCM, and PLR (Fig. 4d–n except 4g) outside of the different improvement regions (Table 2, row 18). It does not interact with the highest improvement region but is the closest near the inferior, anterior medial edge (Fig. 4l, m). Based on this stimulation map, the position + 2 (Fig. 4l) on the central trajectory would be optimal to implant the lead for chronic stimulation (Table 3, row 9).

**Table 2:**
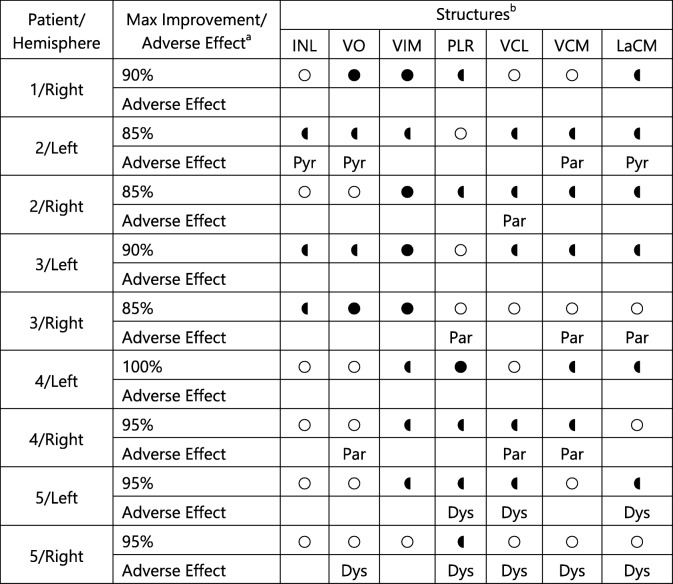
Summary of the different stimulation maps. The interaction of seven structures with the highest improvement region and the adverse effect threshold outline for each implantation is listed

^a^ For the interaction with adverse effect threshold outline, only the anatomical structures which were penetrated by different adverse effect threshold outlines *outside of the therapeutic improvement regions* are considered

^b^where the different symbols are described

**Table 3:** The choice of depth and the trajectory where the distal border of lowest contact of the permanent DBS lead should be implanted based on improvement maps

Patient number	Hemisphere	Optimum implantation depth of permanent lead based on stimulation maps	
1	Right	Central	+ 3 mm
2	Left	Central	− 1 mm
2	Right	Central	+ 1 mm
3	Left	Central	+ 1 mm
3	Right	Central	+ 2 mm
4	Left	Central	+ 4 mm
4	Right	Central	+ 1 mm
5	Left	Central	+ 2 mm
5	Right	Central	+ 2 mm

The procedure described above was applied to all the 9 implantations and the results were summarized. The interactions between the anatomical structures and the highest improvement region and the adverse effect threshold outline respectively are listed in Table 2. Table 3 lists the choice of optimal position and the trajectory for chronic stimulation using the DBS lead for all stimulation maps.

In the 9 stimulation maps, seven anatomical structures were identified that interacted with the highest improvement region: INL, VO, VIM, PLR, VCL, VCM, and LaCM. The VIM, the planned target for all the patients, occurs the most frequently. It appears in 8 out of the 9 maps, with a complete coverage in 4 cases. In 6 of the 9 stimulation maps, the highest improvement region encompassed the posterior subthalamic area (PSA) inferior to the VIM which includes the PLR and the zona incerta (ZI). This area was not explored in the other three maps as adverse effects were observed in this position at low stimulation current amplitudes. In addition, the anterolateral part of the LaCM was partially enclosed in 6 maps, lateral parts of the ventrocaudal medial nucleus (VCM) in 5 maps, the VO was encompassed in 4 maps (2 partially, 2 fully), the inferomedial part of the VCL in 4 maps, and the inferior part of intermediolateral nucleus (InL) was partially included in 3 maps.

Three different types of adverse effects were observed during 6 out of the 9 implantations as described in Table 3: dysarthria, paresthesia, and pyramidal effects. Dysarthria was observed in both implantations of patient 5, and the outline in the two maps (Fig. 4) is posterior and slightly lateral compared with the different therapeutic regions. Adverse effects induced due to stimulation of pyramidal tract were also observed during 2 implantations (patient 2 left and patient 4 right). The stimulation maps of these implantations showed that the adverse effect outlines were superior and lateral to the thalamus. Paresthesia was also observed in 4 implantations (patient 2 left and right, patient 3 right, and patient 4 right). The adverse effect outline for these stimulation maps was observed to be posterior to the VIM and penetrated the VCM, VCL, and LaCM nuclei. In case of paresthesia and dysarthria, VO and PLR are touching the adverse effect threshold outline for some implantations, while in others they are completely enclosed by the highest improvement region without inducing any adverse effects.

## Discussion

This paper describes a new digital approach to assist the clinicians in identifying the optimal region to implant the chronic DBS lead after intraoperative stimulation tests: a task currently performed by evaluating handwritten notes taken during surgery. The approach combines the outline of the relevant anatomical structures with a novel technique to quantitatively evaluate the therapeutic effects and a patient-specific method to estimate the spatial effects of stimulation. This combination creates stimulation maps, i.e., 3D visualization of the intraoperative stimulation test results with therapeutic areas, adverse effect areas, and anatomical structures in the stimulation test region. Nine stimulation maps were generated after applying this approach to 5 ET patients who underwent DBS surgery. In comparison to the current practice of handwritten notes, the interactivity and visualization of maps significantly simplifies the discussion within the surgical team to determine the optimal implant position of the DBS lead. The added information about spatial effect of stimulation with sub-millimeter resolution presented in the stimulation maps enables the implantation of the lead at locations in between the ones that are tested along the trajectory during the surgery.

### Related work

Visualization of intraoperative DBS data has been previously proposed. D’Albis et al. [13] developed PyDBS to automate tasks like image registration, image segmentation, and visualization. They designed it to assist clinicians during pre-operative planning and post-operative electrode validation, but not during intraoperative electrode placement. Guo et al. [20] used non-linear image registration to visualize digitized brain atlas, segmented deep brain nuclei, and final surgical targets as well as electrophysiological information from their own database on the patient images. Although their software could be used during the surgery, it did not have provisions to visualize therapeutic or adverse effects of stimulation at a given position. The group of D’Haese [14] developed a system consisting of a central repository (cranial vault), modules to interact with the repository (CRanial VAult Explorer, CRAVE), and algorithms to automate certain tasks in the DBS treatment (pre-operative, intraoperative, and post-operative phases). Their system is able to visualize therapeutic and adverse effects of stimulation, which the clinical team would manually enter during the surgery. However, the use of their intraoperative module during surgeries showed that the manual task of providing information to their system was stressful for the surgical team. In addition, none of the software described above estimates the spatial effect of stimulation. Miocinovic et al. [32] proposed a software called Cicerone which visualizes patient images with brain atlas, MER, DBS leads, and the volume of tissue activated (VTA) in 3 dimensions and can be used intraoperatively. However, the estimation of VTA is not patient-specific and is based only on the DBS lead. In contrast to the existing literature, the approach described in this study was specifically designed for intraoperative use. It benefits from patient-specific EF simulations to estimate the spatial extent of stimulation as well as quantitative evaluations of induced therapeutic effects by using accelerometry.

### Improvement map types

In the present study, we describe a specific case of improvement maps where each voxel in the stimulation test region is assigned with the maximum value of improvement as measured by the quantitative technique. To the best of our knowledge, no such patient-specific intraoperative approach has been proposed in the literature before. In general, however, the structure of the improvement maps, i.e., the different therapeutic regions and their location, is significantly affected by the choice of the improvement value assigned to a voxel. Theoretically, this choice can be made using computationally simple functions like mean, maximum, etc. or complex functions based on fuzzy logic, weighting function based on distance from trajectory, etc. Importantly though, to the best of our knowledge, there are no methods available that would enable validation of these functions for assigning the improvement values. Therefore, the practical implications of this choice have to be considered. For this study, we aimed at identifying the optimal position to implant the permanent electrode. In consequence, it is necessary to identify the highest improvement region with the least amount of current (less battery consumption), justifying our choice of assigning the maximum improvement to each voxel. Furthermore, the use of maximum function also mimics the current clinical procedure of choosing the smallest current resulting in the highest improvement, which makes it intuitive for a clinician to understand. In addition to the choice of improvement value assigned to a voxel, the input stimulation current amplitude used for the EF simulations also affects the improvement maps. Both these choices depend heavily on the goal set forth before the start of data analysis.

### Anatomical interpretation of stimulation maps

The application of the stimulation map approach to five patients showed how it could assist in choosing the depth for the permanent implantation of the DBS lead (Table 3). In addition, they also show the possibility of improving DBS targeting in general. The interactions of the seven structures presented in Table 2 with the therapeutic and adverse effect regions of the stimulation map concur with the findings of other published research. The highest improvement regions in the different stimulation maps are either in the inferior part of the VIM or in the PSA. The VIM is the gold standard target for the treatment of ET patients and results in optimal therapy for most patients [9]. Stimulation in the PSA has been shown to be effective for treating proximal tremor [26, 33, 35], PD [11], and ET [7, 10, 34], and some researchers argue that it is a better target compared with the VIM [21, 37]. With regard to the adverse effects, those associated with the stimulation of the pyramidal tract were observed for two implantations where the threshold outline in the stimulation maps was very close to the internal capsula, supero-lateral to the thalamus. Dowsey-Limousin [16] reported similar effects during post-operative programming of the implanted pulse generator. The dysarthria threshold outline for both implantations of patient 5 suggests that the stimulation of VCL, VCM, and LaCM may be responsible for it. Similar results were observed by the Reker group [36] using post-operative stimulation tests. In contrast to the two adverse effects discussed above, paresthesia which is commonly observed in VIM DBS procedures, has been associated with different structures by different studies (subthalamic nucleus [45], medial lemniscus [25, 27, 33], PSA [18], and ZI [15]). As described above (Fig. 4) and previously [44], the adverse effect outline can be often found in the PSA region suggesting that stimulation there might cause paresthesia, even if they do not indicate which anatomical structure or fiber tract may be responsible for them. When looking at Table 2, VCL, VCM, and LaCM could be candidates as well. Although VO and PLR are also listed in Table 2 for paresthesia as well as dysarthria effects, they are probably not responsible for these effects as in other implantations; they are completely covered by the therapeutic regions without any adverse effect appearance. The similarities discussed here suggest that with a significantly large data set, the stimulation maps could be used to improve DBS targeting and potentially support in studying the mechanisms of actions of DBS.

### Transferability

In its current form described in this study, the procedure of creating the stimulation maps is very specific to the type of data available from the University Hospital in Clermont-Ferrand. In general, however, the technique is very adaptable to the type of data available. In the absence of pre-operative anatomical outline, the patient images can be co-registered to digitized atlases using open-source software like 3D Slicer [17] and segmented to outline the relevant anatomical structures. Regarding the spatial estimation of stimulation, the EF simulation procedure can be adapted to the type of MR data acquired, the type of exploration electrode used, and the stimulation parameters in a given surgical center. Concerning the use of accelerometers to evaluate tremor, our previous study has shown its advantages over the existing visual method and the relative ease with which it can be added to the surgical procedure without major alterations or loss of patient comfort. For DBS procedures of non-tremulous patients, e.g., Parkinson’s disease patients with rigidity, some quantitative tools to evaluate rigidity exist [29, 38], including ours [43]. These methods, however, are in experimental stage. These situations were considered during the design phase of the stimulation map method. Therefore, for making the maps, the improvement in symptoms estimated using routine clinical scales and subjective methods can also be used. To do so, the surgical team has to predetermine the levels of improvement they want to visualize and to note the stimulation parameters that result in these improvements.

### Limitations

The primary focus of this paper is to describe the method of generating the stimulation maps and to apply it to 9 implantations. Nevertheless, to draw statistically significant conclusions, the approach has to be applied to a larger number of patients. Moreover, in order to establish that the implant position of the lead based on the stimulation maps is optimal, a larger control group study must be performed comparing lead implant position choices made using conventional methods with those made based on the stimulation maps. In terms of drawbacks of individual datasets, the reconstruction of the outline of anatomical structures is limited by the voxel dimension, which can only be improved by using imaging systems with better resolution. Any caveats associated with the quantitative tremor evaluation and EF simulation techniques also affect the stimulation maps. Our previous research has shown that the quantitative tremor evaluation depends on proper acquisition of baseline data before every stimulation test [42]. In the absence of sufficient baseline recording, the baseline data of a previous stimulation test position can be used. With regard to the EF simulations, the use of 0.2 V/mm isolevel represents the activation of neuronal fibers with axonal diameter of 3–4 μm as previously described [6] but does not account for their orientation, i.e., anisotropy, the chemical processes that happen in neurons or the network effects between populations of neurons at the tissue level. To the best of our knowledge, models that account for all such interactions between electrical stimulation and neuronal tissue have not yet been published. On the other hand, to be able to use the images with the highest resolution, image fusion was used in a manner that limited the errors associated with transformation and fusion [50]. Further, we do not consider the effects of brain shift on the position of the electrode based on the observation that the largest shift occurs when the exploration electrode is replaced by the chronic DBS lead. For exhaustive description of the limitations of each method, the readers are advised to refer to the respective literature of each method [2, 23, 31, 42].

### Future work

The stimulation maps presented here were generated and analyzed post-operatively. Steps to generate the stimulation maps in real-time have been identified. The time between pre-surgical tasks (image acquisition and surgical planning) and the actual surgery needs to be utilized for all preparatory steps (extract outline of anatomy, generate the patient-specific brain model, etc.). The accelerometer-based symptom evaluation system has already been enhanced to analyze data in real-time. Additional provisions have to be made to establish communication between the accelerometer recording software and the simulation software to simulate EF in real-time. The algorithms that generate the stimulation maps from the EF simulation files take a maximum of 5 min and can be executed once stimulation tests are completed during the surgery. Once these steps are realized, a larger clinical study will be conducted to confirm the advantages of stimulation maps highlighted in the current paper and to make them available during surgical decision-making after the intraoperative stimulation tests.

A likely long-term application of stimulation maps would be to facilitate the use of directional DBS leads to steer the effects of stimulation in a certain direction. Schüpbach et al. [8, 39, 40] recently studied the challenges that directional stimulation would bring to DBS and indicated that patient-specific visualization techniques (like stimulation maps) will be required to limit alterations to targeting and intraoperative standards. Besides the intraoperative application, stimulation maps provide another tool to better the understanding of the mechanisms of action of DBS. By applying this technique to a large patient cohort, a “stimulation atlas” can be built to study the areas responsible for high improvement as well as adverse effects. For this approach, various functions for assigning improvement values to voxels will have to be investigated. Apart from that, using models that consider the interaction of electrical stimulation with the neuronal tissue at various levels (molecular, cellular, and network) once they are available and comparing information to known anatomy and physiology of the disease, we can learn more about the mechanisms by which DBS alleviates symptoms.

## Conclusion

This paper describes a new technique to generate stimulation maps to summarize and visually analyze data from intraoperative stimulation tests for deep brain stimulation surgery. Data collected from 9 implantations were analyzed with the aim to identify the optimal site to implant the chronic DBS lead. Clinicians found the visualizations intuitive and easy to interpret and to identify the region resulting in highest improvement in tremor. In 7 of the 9 stimulation maps, the highest improvement region was found to be in the PSA in agreement with the scientific consensus. This method has the potential to simplify the surgical team’s task in identifying the ideal implant location of the chronic DBS lead and to facilitate and expedite the use of directional leads in DBS.

## Footnotes

1 Andreuccetti, D., R. Fossi and C. Petrucci, Florence, Italy. (2005). “Dielectric properties of the tissue.” from http://niremf.ifac.cnr.it/tissprop/.
